# Lumbar fusion using a carbon-fiber PEEK pedicle screw system combined with a carbon-fiber PEEK TLIF cage: a prospective, multicenter study

**DOI:** 10.1186/s12891-025-08457-7

**Published:** 2025-03-06

**Authors:** Marcus Eif, Thomas Forster, Christoph Fleege, Francis Kilian, Anne Dorothée Schmitt, Dorothea Daentzer

**Affiliations:** 1 Städtisches Klinikum Görlitz gGmbH, Klinik für Neurochirurgie, Girbigsdorfer Straße 1-3, 02828 Görlitz, Germany; 2 Kantonsspital St. Gallen, Ostschweizer Wirbelsäulenzentrum, Rorschacher Strasse 95, St. Gallen, 9007 Switzerland; 3 Orthopädische Universitätsklinik Friedrichsheim gGmbH, Marienburgstrasse 2, 60528 Frankfurt am Main, Germany; 4 Katholisches Klinikum Brüderhaus Koblenz, Klinik für Wirbelsäulenchirurgie, Kardinal- Krementz-Str. 1-5, 56073 Koblenz, Germany; 5 Klinik für Neurochirurgie, UK S-H, Campus Kiel, Arnold-Heller-Str. 3, Haus 41, 24105 Kiel, Germany; 6 Orthopedic Department, Spine Section, Hannover Medical School, DIAKOVERE Annastift, Anna-von-Borries-Str. 1-7, 30625 Hannover, Germany

**Keywords:** Degenerative disc disease, Spine fusion, Pedicle screw, Carbon fiber, CFR-PEEK, Spondylolisthesis

## Abstract

**Background:**

Carbon-fiber-reinforced polyether ether ketone (CF-PEEK) is a radiolucent, non-metallic implant material used for instrumented lumbar spondylodesis. Clinical studies of pedicle screw systems employing this material, especially for degenerative indications, are scant.

**Methods:**

We conducted a multicenter, prospective clinical study to assess clinical and radiographic outcomes in patients with symptomatic degenerative lumbar disk disease, including degenerative spondylolisthesis treated with a CF-PEEK pedicle screw and a transforaminal lumbar interbody fusion (TLIF) cage system. We followed up the participants for two years postoperatively to collect clinical data (via the Oswestry Disability Index, Core Outcome Measures Index, and Visual Analog Scale), radiographic parameters (functional X-rays) to assess fusion status, and any complications.

**Results:**

In total, 86 patients were recruited. During the study, 21 patients (24.4%) dropped out, including 5 (5.8%) who underwent explantation of the study device(s). At the final follow-up, the fusion rate was 98.6% (95% confidence interval, 92.7–100.0%). All clinical parameters improved significantly. There were no complications potentially attributed to the implant material.

**Conclusions:**

The results demonstrate a fusion rate similar to that of metallic implant systems with the use of a CF-PEEK pedicle screw and a TLIF cage system. Further studies with larger samples are needed to substantiate this finding.

**Trial registration:**

The study was registered at ClinicalTrials.gov (NCT02087267). Date of registration: March 12, 2014.

## Introduction

Pedicle screw fixation is commonly used for spinal stabilization when performing surgery for degenerative, trauma, or tumor [1]. A variety of implants composed of different materials are available, the most popular of which are metals, such as titanium and its alloys [2]. It is essential to be able to reliably perform and evaluate postoperative imaging for diagnostic purposes after lumbar spondylodesis. While titanium implants can provide adequate stabilization to the spine, one disadvantage is their metal-induced artifacts on magnetic resonance imaging (MRI), which result in geometric distortion, signal loss, and pile-up artifacts [3]. For many years, carbon-fiber-reinforced polyether ether ketone (CF-PEEK) has achieved positive results in the orthopedic field [4] as well as in spinal fusion techniques [5–8]. The radiolucency of CF-PEEK reduces artifacts on computed tomography (CT) and MRI [9, 10], which makes it better suited for patient follow-up analysis. Metal fiducial markers ensure sufficient radiological visibility, which is advantageous both intra- and postoperatively. In oncological patients, moreover, this material creates less radiation interference than titanium [11, 12], which enables more precise radio or proton therapy.

CF-PEEK is a thermoplastic composite biomaterial that exhibits properties suitable for load-bearing orthopedic implants [13]. This material is composed of 55% carbon fibers and 45% PEEK matrix, which together account for its excellent mechanical and fatigue properties. Surgeons have used CF-PEEK for years in other orthopedic applications, such as intervertebral cages and osteosynthesis plates.

Previous cadaver and clinical studies have shown that CF-PEEK pedicle screw systems are equal to titanium devices in terms of withstanding loads and resisting motion for lumbar fusion [14]. In cases of primary and metastatic spinal tumors, the use of CF-PEEK instrumentation was associated with a low rate of intra- and postoperative complications [15].

While the clinical and radio-oncological advantages of CF-PEEK implants have been shown for tumor patients during their entire courses of therapy, only a single pilot study has described the clinical outcomes in patients requiring arthrodesis of the lumbar spine for degenerative disc disease [16]. Therefore, we conducted this study to evaluate the first prospective series of patients with symptomatic degenerative disc disease, including degenerative spondylolisthesis, who we treated with CF-PEEK instrumentation using a CF-PEEK pedicle screw and a transforaminal lumbar interbody fusion (TLIF) cage system.

## Materials and methods

We report on an international, multicenter, prospective, post-market clinical study to record the outcomes of using a pedicle screw system in combination with a TLIF cage, both composed of CF-PEEK. We conducted the investigation in the daily practice settings of six neurosurgical or orthopedic centers in Germany and Switzerland between March 2014 and January 2019. The inclusion criteria required all patients to have acute or chronic instabilities or deformities of the lumbar and lumbosacral spine as a result of degenerative disc disease and to have been conservatively treated for at least six months.

The exclusion criteria ruled out patients with more than two affected levels, treatments not between L2 and S1, spondylolisthesis classified as Meyerding grade 3 or higher, previous lumbar spinal surgery other than discectomy at the level(s) to be operated on, previous or current infection in the spine or disc, tumors, previous or current illicit drug abuse, or current abuse of alcohol. We excluded prisoners and patients younger than 18 years or older than 79 years from the study. Furthermore, severe osteoporosis or similar bone density loss, including any metabolic bone disease, ruled out participation.

**Fig. 1:**
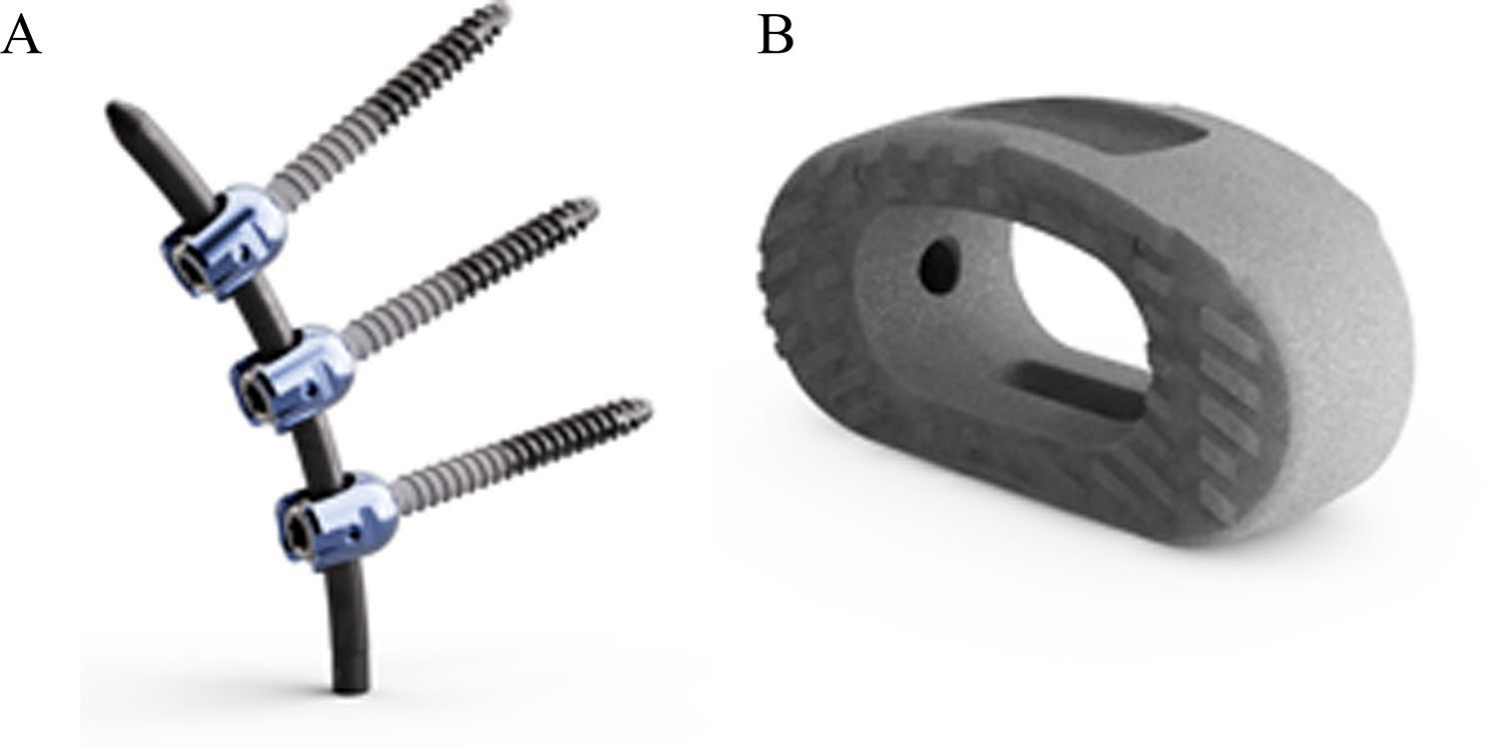
The icotec carbon-fiber PEEK system. (**A)**: The icotec pedicle screw system. (**B**): The icotec ETurn TLIF cage

**Fig. 2:**
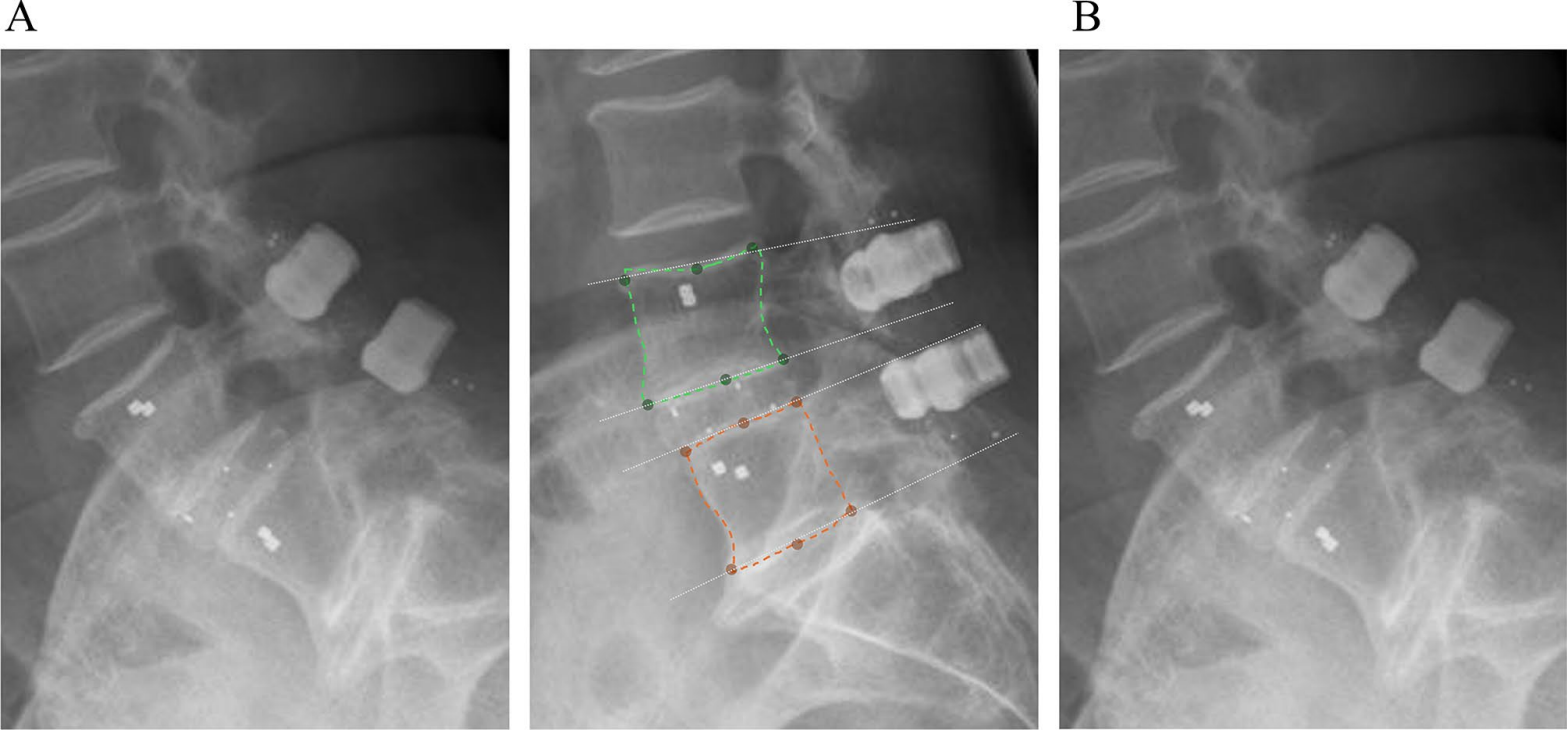
Methodology for measuring the range of motion of vertebrae. (**A**) In the extension image, the upper (green) and lower (orange) vertebrae are identified and control points are generated. (**B**) The control points are transferred to the flexion image using an AI-based registration method. The angles between the vertebrae (lower endplate of cranial vertebral body, upper endplate of caudal vertebral body) are determined based on the control points (white arrow), and the difference yields the range of motion

**Fig. 3:**
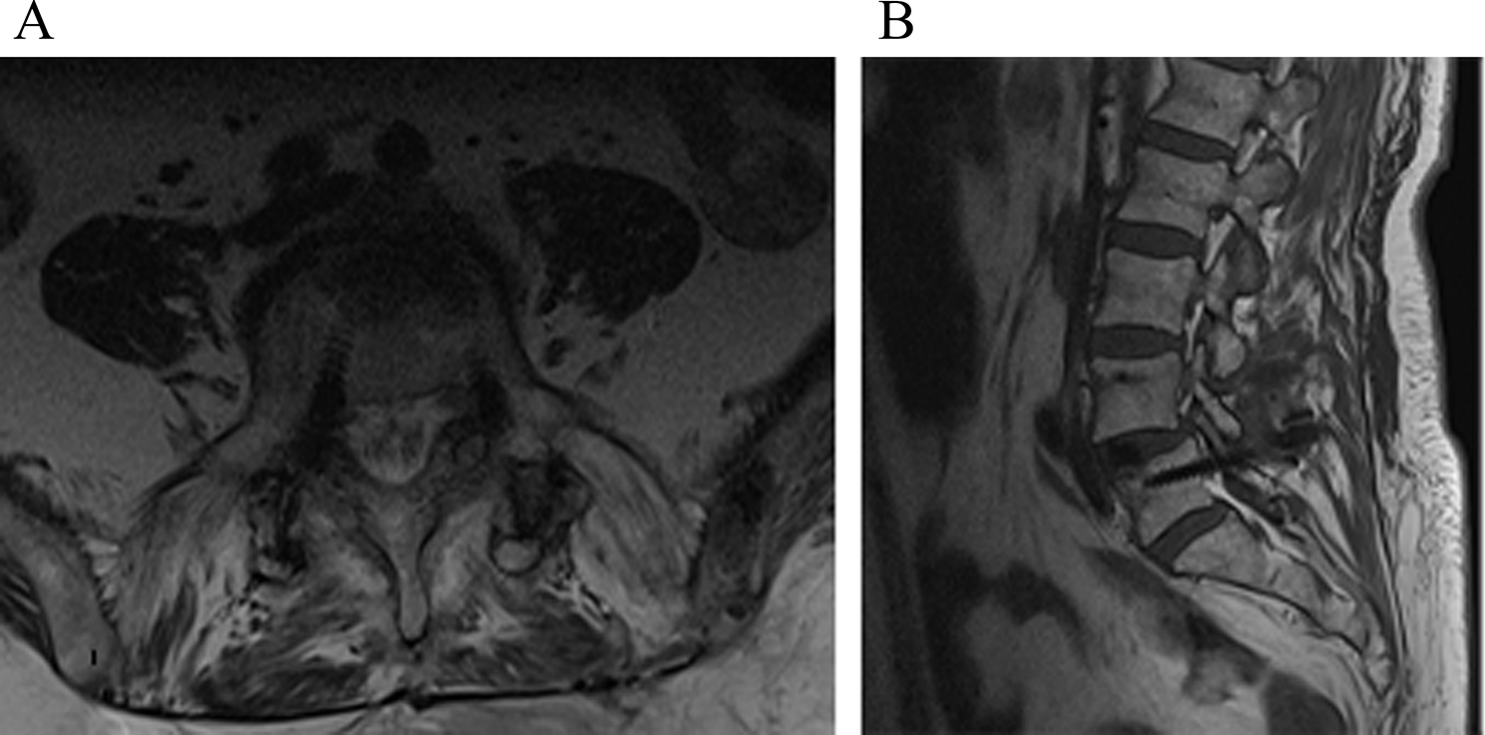
Axial and sagittal views demonstrating the medial positioning of the right L5 pedicle screw following revision surgery. (**A**): Axial view showing the medial deviation of the right L5 pedicle screw. (**B**): Sagittal view illustrating the screw’s position outside the pedicle

For the study, we used the icotec pedicle screw system and the icotec ETurn TLIF cage. Polyaxial cannulated screws were applied in the most common sizes: lengths from 30 to 60 mm and diameters of 5.5, 6.5, and 7.5 mm. The CF-PEEK screw shafts are partially coated with titanium vacuum plasma spray (VPS) (Fig. 1A). This titanium VPS coating leads to increased surface roughness, which allows the bone in the pedicle to achieve better attachment to the screw shaft [13]. The locking caps and tulips are manufactured from titanium alloy (Ti-6Al-4 V ELI). We used straight or pre-bent CF-PEEK rods with a diameter of 5.5 mm and ranging in length from 30 to 100 mm in 10-mm increments.

The icotec ETurn TLIF cage (Fig. 1B) includes a kidney-shaped body with a central window, a distraction-insertion wedge, and treads on the cranial and caudal surfaces that act to guide and anchor the implant. The concentrically positioned treads allow the implant to turn into the disc space to the transverse end position. The treads create a markedly larger pressure-bearing contact surface between the implant and the bone compared to conventional pointed anchoring elements. The cages are available in heights of 7, 9, 11, 13, and 15 mm and in widths of 12 and 16 mm. The cages have a titanium VPS coating to improve direct bone ongrowth [13].

Biomechanical testing, including all relevant ASTM testing, was conducted to evaluate the performance of the pedicle screw system and TLIF cage. These tests assessed the system’s biomechanical properties, including the strength of the rod-screw connection and the contact area of screws and connecting rods with titanium nail tails.

All procedures in this study were performed without the assistance of surgical navigation. All pedicle screws were inserted under fluoroscopic control. We utilized a k-wire introduced into the cannulation of the pedicle screw, allowing for real-time assessment of screw position during intraoperative x-ray imaging. This method provided immediate feedback on the accuracy of screw placement. Postoperatively, the assessment of implant status and potential failures due to radio transparency was conducted through various means. Clinical signs such as pain, restricted movement, or neurological deficits prompted further investigation. Additionally, advanced imaging modalities such as CT scans were used for a detailed evaluation of the implant position and integrity.

Imaging of the patients was performed following the schedule presented in Table 1.

**Table 1:** Overview of assessments performed for the study

	Preoperative	1–5 days	3 months	6 months	12 months	24 months
Neutral standing a/p and lateral radiography	X	X	X	X	X	X
Active flexion and extension radiography	X*	-	X*	X	X*	X
CT or MRI	X	-	-	-	-	-
MRI	-	-	-	-	-	X
Clinical follow-up	X	-	X	X	X	X

* Optional. Abbreviations: a/p, anteroposterior; CT, computed tomography; MRI, magnetic resonance imaging

The primary outcome parameter of segmental fusion was evaluated by measuring the disc angle between the lower endplate of the cranial vertebra and the upper endplate of the caudal vertebra. We determined angular motion based on functional radiographs as described by Schulze et al. [17] and reported it in units of degrees. The system demonstrates high accuracy in measuring angular motion, with a mean error of 0.04° ± 0.13° [17]. Angular motion was defined as the difference in disc angles on lateral functional radiographs from full extension to full flexion at each instrumented level per follow-up examination. Disc height was defined as the average of the anterior and posterior disc height at each instrumented level on neutral lateral radiographs.

Two independent radiographic reviewers performed all additional radiographic evaluations. They had been trained in the classification systems used in this study as well as in the design and radiographic features of the device under investigation. The reviewers were blinded to the clinical outcomes of the patients. We identified successful overall fusion if there was evidence of bridging trabecular bone between the upper and lower vertebral endplates and angular motion less than 5°, translational motion less than 3 mm, and evidence of left and/or right posterolateral bridging bone between the facet joints if fused (Fig. 2) [18]. For two-level involvement, both levels had to be fused for the fusion to be considered successful.

Patients were clinically examined, and data were recorded before surgery, at surgery and at 3, 6, 12, and 24 months after surgery. Clinical assessments at each interval drew on the Visual Analog Scale (VAS) to measure lower-back pain and leg and buttock pain (experienced in the pain-dominant leg) as well as the patient’s satisfaction with the surgery and documentation of complications. Additional clinical data were recorded, including implant failures, surgery-related intraoperative, postoperative, and general complications and subsequent surgical interventions.

This study was performed in accordance with relevant guidelines and regulations. Ethics committee approval was obtained prior to commencing the study (Freiburger Ethik-Kommission, approval number 014/1175). All subjects provided written informed consent.

A prior sample size calculation was performed based on a non-inferiority hypothesis by means of the primary study endpoint (i.e., fusion). Assuming a fusion rate of 94%, a dropout rate of 20%, and a value of α set at 5%, the required sample size for the desired 95% confidence interval (CI) of ± 5% was *n* = 86 (BiAS for Windows, epsilon-Verlag Hochheim Darmstadt, Germany). The null hypothesis of the study stated that fusion would be 89% or less at the final follow-up.

Continuous variables were expressed as mean ± standard deviation and categorical variables as absolute counts and/or percentages. A last-observation-carried-forward approach was applied to the primary endpoint fusion in those subjects in which no evaluation of fusion was possible at two years after surgery.

## Results

At the conclusion of the study 86 patients at 6 study centers had been prospectively operated on with pedicle screws and cages from the icotec system. The mean age of the study population was 58.2 ± 13.7 years (range: 20–80 years). We included 50 females (58.1%) and 36 males (41.9%). The mean body mass index was 27.5 ± 4.3 kg/m^2^ (range: 19.1–43.7), and 29 (33.7%) patients were smokers. Overall, 95 segments (L3–L4 (*n* = 6), L4–L5 (*n* = 64), and L5–S1 (*n* = 25)) were treated. A total of 77 patients (89.5%) received monosegmental and 9 patients (10.5%) bisegmental instrumentation. A total of 362 screws were used.

No screening failures were noted. During the course of the study 6 patients (7.0%) were lost to follow-up, 2 (2.3%) died from unrelated causes, 7 (8.1%) withdrew their consent, 1 (1.2%) withdrew due to morbidities unrelated to the procedure, and 5 (5.8%) underwent revision surgery due to use error, for corpectomy due to osteoporosis, to lengthen the system (> 2 levels), for cement augmentation of the screws, and to implant an intermediate (8-mm) cage size from another manufacturer, respectively. Therefore, 65 patients completed all follow-ups for the study. In addition, fusion information from 10 of the censored patients was carried forward from an earlier follow-up visit and could therefore be evaluated.

In 74 out of 75 patients, we could radiographically establish fusion. The fusion rate, therefore, was 98.7% (95% CI, 92.8–100.0%). Hence, based on the 95% CI, the findings refuted the null hypothesis of inferiority (i.e., a fusion rate of 89% or less).

Transforaminal lumbar interbody fusion reduced the range of angular motion by 3.2° on average and anteroposterior translation by 0.5 mm on average. Furthermore, the initial intervertebral disc height correction of 1.7 mm could not be maintained over time (Table 2).

**Table 2:** Radiographic outcomes

	Angular range of motion [°] ^§^	AP translation [mm] ^§^	Intervertebral disc height [mm] ^§^
Preoperative	4.0 ± 4.0 (0.1–14.7)	0.8 ± 0.7 (0.1–3.0)	6.3 ± 2.3
1–5 days	-	-	8.0 ± 1.9
3 months	1.6 ± 1.5 (0.0–7.1)	0.5 ± 0.5 (0.0–2.2)	6.9 ± 1.8
6 months	1.3 ± 1.3 (0.0–5.7)	0.4 ± 0.5 (0.0–2.3)	6.5 ± 2.1
12 months	0.8 ± 0.6 (0.0–2.4)	0.3 ± 0.3 (0.0–1.2)	6.4 ± 1.9
24 months	0.8 ± 0.8 (0.0–4.1)	0.3 ± 0.3 (0.0–1.0)	6.1 ± 1.9

§ Expressed as mean ± standard deviation (range)

The VAS for back pain decreased from 63.5 ± 22.1 preoperatively to 28.4 ± 26.2 at 24 months (*p* < 0.001), while for leg pain, it declined from 63.2 ± 22.0 preoperatively to 25.3 ± 25.8 at 24 months (*p* < 0.001). The mean Oswestry Disability Index (ODI) score decreased from 49.9 ± 15.4 to 23.0 ± 18.7 at 24 months (*p* < 0.001), and the mean Core Outcome Measures Index (COMI) declined from 7.8 ± 1.4 points to 3.1 ± 2.4 points (*p* < 0.001). Patient satisfaction (on a VAS from 0 to 100) was 77.5 ± 27.0 at 24 months (Table 3).

**Table 3:** Clinical outcomes

	Preoperative	3 months	6 months	1 year	2 years
ODI	49.9 ± 15.4	29.4 ± 18.0	22.0 ± 16.8	22.4 ± 16.1	23.0 ± 18.7
COMI	7.8 ± 1.4	4.0 ± 2.6	3.0 ± 2.3	3.1 ± 2.4	3.1 ± 2.4
Back pain (VAS)	63.5 ± 22.1	31.7 ± 23.4	23.6 ± 21.9	22.0 ± 22.1	28.4 ± 26.2
Leg pain (VAS)	63.2 ± 22.0	23.9 ± 24.9	23.9 ± 24.9	21.5 ± 23.6	25.3 ± 25.8
Satisfaction (VAS)	-	79.6 ± 24.5	83.8 ± 22.6	85.4 ± 19.1	77.5 ± 27.0

All values expressed as mean ± standard deviation; Abbreviations: ODI, Oswestry Disability Index; COMI, Core Outcome Measures Index; VAS, Visual Analog Scale

A total of 42 adverse events were reported (Table 4).

**Table 4:** Summary of adverse events during the entire follow-up period

Complication	Occurrences (number of observations)		
	Intraoperative	Early postoperative (≤ 30 days)	Late postoperative (> 30 days)
**General**			
Cerebrovascular incident	-	1	1
Cardiovascular	-	1	1
Urinary tract infection	-	1	-
**Device- or procedure-specific**			
Dura lesion	2	-	1
Adjacent segment pathology	-	1	2
Disc herniation / sequestration	-	2	-
Dysesthesia	-	1	-
Cage subsidence	-	1	1
Cage migration	-	2	4
Hematoma	-	2	-
Seroma	-	1	-
Pain	-	3	4
Pedicle fracture	-	1	-
Screw malpositioning	-	1	-
Screw loosening	-	-	4
Cyst	-	-	1
Sacroiliac joint pathology	-	-	1
Radiculopathy	-	-	1
Calcification	-	-	1

Among the 42 adverse events documented, 15 (35.7%) were categorized as implant-related, necessitating reoperation (Table 5). The incidence of implant-related events leading to reoperation was 17.4% (i.e., 15 cases in 86 patients). Importantly, none of these reoperation cases were attributed to the implant design or material. Notably, screw malplacement was observed in one patient (Fig. 3), while the remaining cases were part of the broader category of implant-related events leading to reoperation. We could not attribute any neurological complications to screw malpositions or any types of complications to the specific devices or CF-PEEK material used.

**Table 5:** Overview of complications leading to reoperation

Case number	Description
1	Repositioning of a misplaced (but non-symptomatic) screw, four days postoperative
2	Removal of sequestrum and decompression at index level, four days postoperative
3	Removal of the pedicle system and cage L4/L5, three months postoperative
4	Replacement of loose screws and malpositioned screws, four weeks postoperative
5	Neurolysis, osteochcondritic segment with bulging annulus, four days postoperative
6	Removal of malpositioned S1-screw, four months postoperative
7	Decompression and stabilization of adjacent level, nine days postoperative
8	Removal of the system due to spondylitis and non-union, three months postoperative
9	Cage revision, 2 mm higher cage, three months postoperative
10	Revision of migrated cage (patient non-compliance), six weeks postoperative
11	Extension of fixation cranial 1 level, two days postoperative
12	System replacement due to spontaneous fusion of adjacent level, eight weeks postoperative
13	Repositioning of misplaced (but non-symptomatic) screw, three days postoperative
14	Repositioning of migrated (but non-symptomatic) cage, three days postoperative
15	Removal of intervertebral cage, replaced by vertebral body replacement device following its subsidence into osteoporotic bone. Implantation of a long titanium construct with cement augmentation, two days postoperatively

## Discussion

Our study confirms the findings of Ghermandi et al., who reported an 89% fusion rate (with an inferred 95% CI, 71.7–97.7%) and significant clinical improvement at 1 year follow-up in patients treated with a CF-PEEK pedicle screw and a PEEK core/titanium-surfaced interbody cage system [16]. Our study confirms their findings with a higher fusion rate (98.6%) and a more robust assessment of the CF-PEEK pedicle screw’s effectiveness in clinical practice, as evidenced by our larger sample size and multicenter setting.

The radiographic results of the study showed that the rate of fusion in spondylodesis of the lumbar spine using a CF-PEEK implant system is non-inferior to the fusion rate obtained with metallic implant systems. A recently published meta-analysis reported a pooled fusion rate of 93.9% (95% CI, 86.6–98.5%) for metallic fusion implants with autologous bone [19], which serves as a benchmark for the findings of this study. The high fusion rate shown in our investigation might be attributed to two properties of the CF-PEEK implants: a lower implant stiffness compared to metal systems and the rough titanium coating, which leads to fast and reliable bone attachment and thus supportive fusion.

Compared with titanium and stainless steel, CF-PEEK has a modulus of elasticity closer to that of bone [20]. Therefore, it has been asserted that CF-PEEK screw/rod structures better approximate the normal biomechanics of the spine via better load distribution across the anterior spine and may consequently promote spinal fusion [21, 22]. However, we did not set out to assess the validity of this statement. Previous research has demonstrated that the rough titanium coating provides excellent bone attachment [13], and it proved to have a positive effect in the clinical setting of this study. Another potential advantage of using CF-PEEK composites for screw/rod constructs is that they can overcome issues related to the radiographic imaging of systems made with standard stainless steel or titanium [9, 10, 23]. Postoperative neurological deficits following instrumented lumbar spondylodesis are possible, and metal implants can cause uncertainty in MRI analyses; for example, the difference between hematoma and residual stenosis may not be clear in a radiologic assessment. Also, it is easier to evaluate the spinal canal and neuroforamina in the presence of implants made of material with lower magnetizability [10]. This imaging advantage plays a crucial role in all indications for which the device is used. In tumor patients, MRI allows optimal assessment, i.e., surveillance of local tumor progression and early detection of recurrences. For degenerative patients, more accurate imaging and interpretation may lead to more specific management of any complications that may occur in the postoperative phase.

In our study, the clinical postoperative outcomes were favorable. The mean improvement in ODI and back and leg pain decisively exceeded the threshold of the minimally clinically important difference, which has been reported to be 12.8 points for ODI, 1.2 points for back pain, and 1.6 points for leg pain [24].

The intervertebral disc height correction of 1.7 mm was not found to be sustainable, as we found a significant correction loss during the early follow-up period. However, in our study, the correction loss did not progress over time, and we did not observe subsequent radicular symptoms following the loss of disc height.

The number of adverse events reported appears to be relatively high. Because an independent contract research organization monitored this prospective study, we recorded all adverse events, including those related to the overall morbidity of this (partly frail) population. This finding reflects the real-world results at the participating institutions and is not specific to the implant material or design.

## Limitations

The current study has several limitations. The dropout rate was relatively high. However, we imbued the investigation with adequate power to refute the null hypothesis. A limitation of all uncontrolled studies is that they carry a risk of selection bias in addition to the risk of confounders, and it is therefore advisable to interpret causal inferences from this study with caution. Nonetheless, the present study counteracted the criticism directed toward uncontrolled studies by consecutively enrolling patients and prospectively documenting the database in relation to the clinical and radiological findings.

Another limitation may be the use of functional radiographs to determine fusion. Currently, the literature specifies no generally accepted fusion criteria. Thin-section multidetector-row CT is considered the gold standard for evaluating fusion, but most surgeons still do not perform this procedure [25]. Although functional radiography is an established method of determining fusion [26, 27], its use is not without controversy. We used the 5° cutoff described by the United States Food and Drug Administration. However, it has been shown that this method leads to an overestimation of the fusion rate compared with thin-section helical CT [28]. Nonetheless, comparing the fusion rate to published values for a 2° cutoff, this study demonstrated a high rate of 98.7% at 24 months compared to 74% at 60 months in Santos et al. [28].

In conclusion, this study shows that using a CF-PEEK implant system to treat degenerative lumbar disc disease leads to a fusion rate similar to that of metallic implant systems. The patients’ quality of life improved in both the immediate and long-term postoperative periods. Therefore, CF-PEEK may be an alternative to metal implant systems, especially for patients who could benefit from the diagnostic advantages offered by this material. However, further studies with larger sample sizes that directly compare the CF-PEEK implant system to metal implant systems are needed to support this claim.

## Data Availability

The datasets analyzed during the current study are available from the corresponding author on reasonable request.
